# The near-complete genome of coxsackievirus A16 genotype B1c causing hand, foot, and mouth disease in Thailand, 2023

**DOI:** 10.1128/mra.01354-24

**Published:** 2025-07-02

**Authors:** Kittisak Taoma, Pichamon Sittikul, Juthamard Chuaysri, Pimolpachr Sriburin, Warisa Nuprasert, Jittraporn Rattanamahaphoom, Nathamon Kosoltanapiwat, Pornsawan Leaungwutiwong, Janjira Thaipadungpanit, Supawat Chatchen

**Affiliations:** 1 Department of Tropical Pediatrics, Faculty of Tropical Medicine, Mahidol University 115374, Bangkok, Thailand; 2 Department of Clinical Tropical Medicine, Faculty of Tropical Medicine, Mahidol University 115374, Bangkok, Thailand; 3 Department of Microbiology and Immunology, Faculty of Tropical Medicine, Mahidol University 115374, Bangkok, Thailand; 4 Mahidol-Oxford Tropical Medicine Research Unit, Faculty of Tropical Medicine, Mahidol University 115374, Bangkok, Thailand; Katholieke Universiteit Leuven, Leuven, Belgium

**Keywords:** pediatric infectious disease, enterovirus, HFMD, coxsackievirus A16, Thailand

## Abstract

We report the near-complete genome sequences of the human Enterovirus strain AI005 isolated from a stool sample of a hand, foot, and mouth disease patient in Thailand. Sequence analysis showed that the genome of isolate AI005 belonged to the CVA16 genotype B1c (genus *Enterovirus*, family Picornaviridae) reported from Thailand.

## ANNOUNCEMENT

Hand, foot, and mouth disease (HFMD) is caused mainly by enteroviruses belonging to the species *Enterovirus alphacoxsackie* of the genus *Enterovirus*, family Picornaviridae (1). Enterovirus A71 and coxsackievirus A16 (CVA16) have been the major viruses causing HFMD for decades (2). The latter virus possesses a positive-sense single-stranded RNA containing approximately 7,410 nucleotides (nt) with a single open reading frame (ORF) of 6,579 nucleotides and 2,193 amino acids, flanked by 5′ and 3′ untranslated regions (UTR) (3). The ORF is composed of three protein precursors: P1, P2, and P3 (3). CVA16 comprises four genotypes, denoted A–D. In Thailand during 2008–2011, the whole-genome sequence of CVA16 genotype B1a isolated from children with HFMD disease was reported (1). During 2000–2022 in Thailand, only CVA16 belonging to genotypes B1a and B1b were reported (2, 4), while CVA16 subgenotype B1c was found circulating in India (5, 6) and China (7).

We report the near-complete genome sequence of CVA16 genotype B1c strain AI005, isolated from the stool of a male HFMD-infected child in Bangkok, Thailand, in August 2023. The patient participated in a study approved by The Ethics Committee of the Faculty of Tropical Medicine, Mahidol University (protocol no. TMEC 23-042), and Institutional Biosafety Committee, Faculty of Tropical Medicine, Mahidol University (protocol no. FTM-IBC 24-30). The stool sample was inoculated onto Vero cells for virus isolation (8). Viral RNA was extracted from the supernatant by the Quick-RNA Microprep Kit (Zymo Research, Irvine, CA, USA) and then amplified using sequence-independent, single-primer amplification method, a widely used technique for viral whole-genome sequencing (9, 10). The nanopore sequencing libraries were prepared using the Ligation Sequencing Kit (SQK-NBD114.96) and sequenced on Oxford Nanopore MinION Mk1B using R10.4.1 flow cells (FLO-MIN114) (Oxford Nanopore Technologies, Oxford, UK).

Base calling was performed using Dorado version 7.4.14 in high accuracy mode. A total of 152,962 raw reads were generated with N_50_ of 527. Enterovirus reads were selected using a BLASTx-based aligner, resulting in the remaining 19,808 enterovirus-specific reads with N_50_ of 697. The ensemble-based genome assembly pipeline, based on a *de novo* assembly approach, used for this analysis is available on GitHub (https://github.com/Ktaoma/entero01). The consensus genome was generated with average depths of 1697.87× and GC contents of 47.3%.

**Fig 1:**
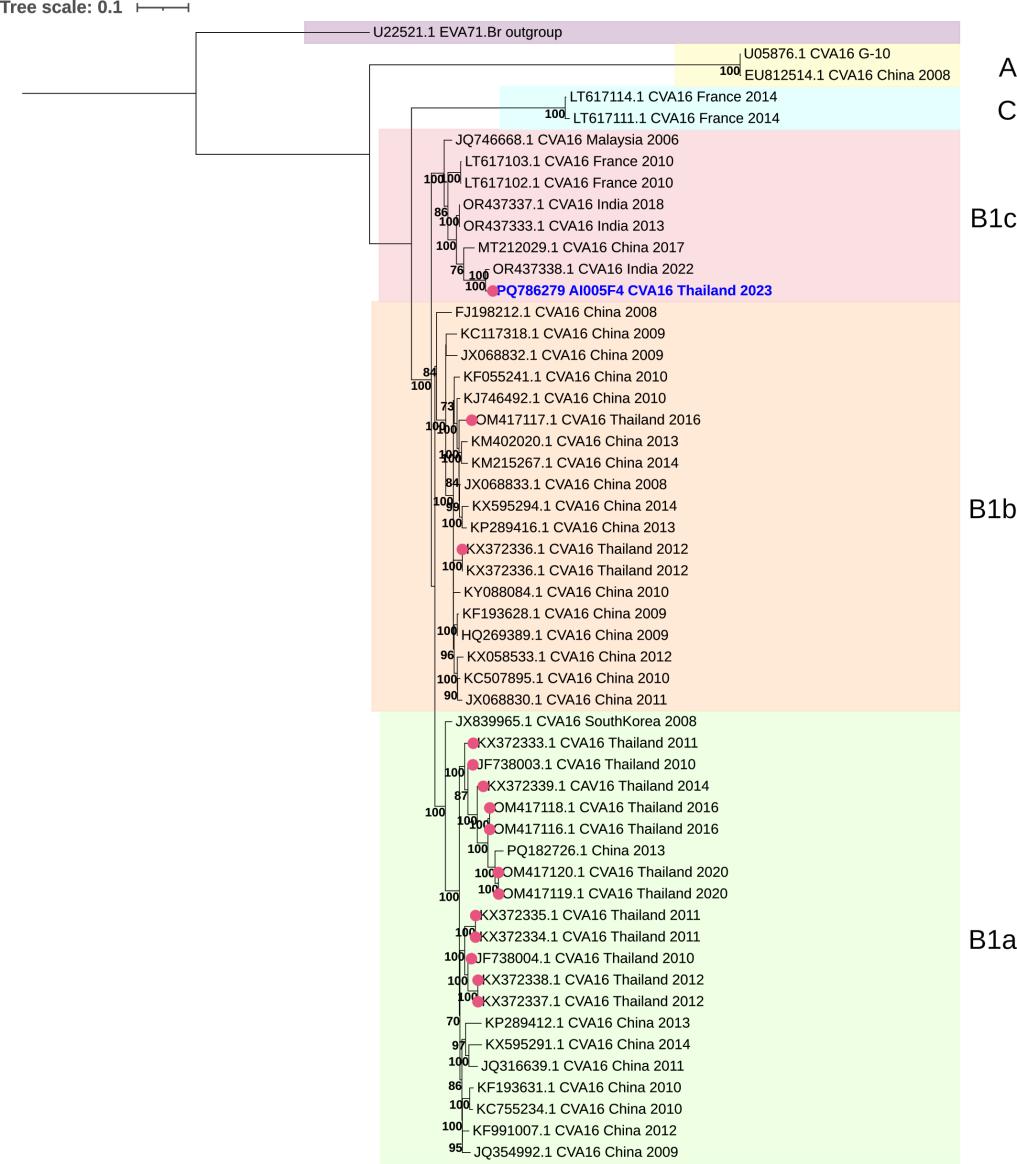
The phylogenetic tree of the CVA16 near-complete genome was constructed by the maximum likelihood method based on the general time-reversible model for 1,000 bootstrap replicates using molecular evolutionary genetics analysis (MEGA 11) software (11). Bootstrap values (>80%) are shown at the branch. The phylogenetic tree was annotated using Interactive Tree Of Life (12). Sequences used in the phylogeny were retrieved from GenBank (black color), and accession numbers, country, and reported year are indicated. The CVA16 strain AI005 sequence is shown in blue color. The red dot represents the CVA16 isolated from Thailand.

The CVA16 strain AI005 contains 7,350 nucleotides in length, excluding the poly(A) tail. The 5′ UTR region contains 716 bases (nt 1–716), followed by an open reading frame nt 717–7,298, and the 3′ UTR (51 bases, nt 7,299–7,350). The AI005 sequence was subjected to BLASTn analysis, and it was found to be closely related to that of CVA16 genotype B1c collected in India in 2022 (accession no: OR437338.1) with about 98% nt identity of the complete genome. The phylogenetic tree analysis of CVA16 was shown in Fig. 1. This study demonstrates that the CVA16, B1c genotype also appeared in Thailand, but it is unknown whether it is an imported or a local epidemic strain.

## Data Availability

The near-complete genome sequence of the CVA16 strain AI005 isolate has been deposited in GenBank under the accession number PQ786279.1. Raw sequencing reads were deposited in the Sequence Read Archive (SRA run: SRR32105493) under BioProject accession number PRJNA1214984.
